# Construction and Validation of a Ferroptosis-Related Prognostic Model for Gastric Cancer

**DOI:** 10.1155/2021/6635526

**Published:** 2021-02-28

**Authors:** Xiaotao Jiang, Qiaofeng Yan, Linling Xie, Shijie Xu, Kailin Jiang, Jiahua Huang, Yi Wen, Yanhua Yan, Junhui Zheng, Shuting Tang, Kechao Nie, Zhihua Zheng, Jinglin Pan, Peng Liu, Yuancheng Huang, Xingrui Yan, Yushan Zou, Xuan Chen, Fengbin Liu, Peiwu Li, Kunhai Zhuang

**Affiliations:** ^1^Department of Gastroenterology, The First Affiliated Hospital of Guangzhou University of Chinese Medicine, Guangzhou 510405, Guangdong, China; ^2^First Clinical Medical College, Guangzhou University of Chinese Medicine, Guangzhou 510405, Guangdong, China; ^3^No. 1 Traditional Chinese Medicine Hospital in Changde, Changde 415000, Hunan, China; ^4^Guangzhou University of Chinese Medicine, Guangzhou 510405, Guangdong, China; ^5^Department of Gastroenterology, Hainan Provincial Hospital of Traditional Chinese Medicine, Haikou 570100, Hainan, China; ^6^Ningde Hospital of Traditional Chinese Medicine Affiliated to Fujian University of Traditional Chinese Medicine, Ningde 352100, Fujian, China; ^7^Baiyun Hospital of The First Affiliated Hospital of Guangzhou University of Chinese Medicine, Guangzhou 510470, Guangdong, China; ^8^Lingnan Medical Research Center, Guangzhou University of Chinese Medicine, Guangzhou 510405, Guangdong, China

## Abstract

**Background:**

Gastric cancer (GC), an extremely aggressive tumor with a very different prognosis, is the third leading cause of cancer-related mortality. We aimed to construct a ferroptosis-related prognostic model that can be distinguished prognostically.

**Methods:**

The gene expression and the clinical data of GC patients were downloaded from The Cancer Genome Atlas (TCGA) and Gene Expression Omnibus database (GEO). The ferroptosis-related genes were obtained from the FerrDb. Using the “limma” R package and univariate Cox analysis, ferroptosis-related genes with differential expression and prognostic value were identified in the TCGA cohort. Last absolute shrinkage and selection operator (LASSO) Cox regression was applied to shrink ferroptosis-related predictors and construct a prognostic model. Functional enrichment, ESTIMATE algorithm, and single-sample gene set enrichment analysis (ssGSEA) were applied for exploring the potential mechanism. GC patients from the GEO cohort were used for validation.

**Results:**

An 8-gene prognostic model was constructed and stratified GC patients from TCGA and meta-GEO cohort into high-risk groups or low-risk groups. GC patients in high-risk groups have significantly poorer OS compared with those in low-risk groups. The risk score was identified as an independent predictor for OS. Functional analysis revealed that the risk score was mainly associated with the biological function of extracellular matrix (ECM) organization and tumor immunity.

**Conclusion:**

In conclusion, the ferroptosis-related model can be utilized for the clinical prognostic prediction in GC.

## 1. Introduction

According to the latest global cancer statistics, gastric cancer (GC) ranks fifth in the incidence of cancers and is the third leading cause of cancer-related mortality [1]. The overall survival of patients with GC varies widely in different regions of the world. For example, the 5-year survival rate is 31% in the United States, 19% in the United Kingdom, and 26% in Europe [1]. The conditions mentioned above indicated that GC is a disease with high heterogeneity [2]. The conventional system for prognosis prediction, such as histological grade and tumor stage, is becoming increasingly difficult to cover the clinical diversity of GC [3]. Therefore, developing a novel prognostic model is urgent.

Cancer cells often have defects in the execution of cell death, which is one of the main reasons leading to treatment resistance [4]. Ferroptosis is a newly discovered form of regulating cell death, which was driven by the accumulation of lipid peroxidation and lethal reactive oxygen species [5]. In recent years, ferroptosis has gradually become a promising therapeutic method for inducing cancer cell death [6]. Ferroptosis has been observed associated with tumor prognosis [7] and a prognostic ferroptosis-related gene signature has been successfully established in hepatocellular carcinoma [8]. In GC, some genes have been found to play a vital role in regulating ferroptosis. For example, CDO1 [9] and ALOX15 [10] can promote ferroptosis in human GC cell while SCD1 [11], PLIN2 [12] and GDF15 [13] are opposite. However, whether these ferroptosis-related genes are correlated with the prognosis of GC patients still remains to be explored.

Herein, we constructed a ferroptosis-related prognostic model based on mRNA expression profiles and clinical data of GC patients from TCGA and validated it in a meta-GEO cohort. Analyses of functional enrichment and tumor microenvironment were also conducted to explore the potential mechanisms.

## 2. Materials and Methods

**Figure 1:**
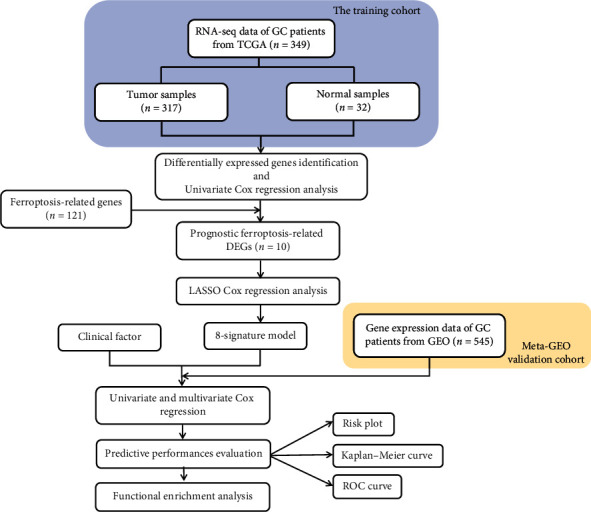
The flow diagram of data collection and analysis in the present study.

The flowchart of this research is shown in Figure 1.

### 2.1. Data Collection and Preprocessing

The gene expression and corresponding clinical information of stomach adenocarcinoma (STAD) samples were downloaded from the UCSC Xena browser (https://xenabrowser.net/) [14]. 375 STAD samples with expression and clinical data were obtained. After removing samples with 0-days follow-up duration and incomplete clinical information, 317 STAD samples and 32 adjacent samples were obtained and used for the primary cohort. Gene expression data and corresponding clinical information of GSE66229 [15], GSE15459 [16], and GSE34942 [17] datasets, totally including 556 GC patients, were retrieved from the Gene Expression Omnibus (GEO) database. The above three GEO datasets were performed on the same microarray platform of [HG-U133_Plus_2] Affymetrix Human Genome U133 Plus 2.0 Array. After removing 11 patients with incomplete clinical information or 0-days follow-up duration, the remaining 545 GC patients were consolidated as a GC meta-GEO cohort. The batch effects were removed by the “combat” function of the “sva” package of R. Finally, 317 GC patients from the TCGA-STAD cohort and 545 GC patients from the meta-GEO cohort were enrolled. The clinical characteristics of the patients above are detailed in Table 1. FerrDb (http://www.zhounan.org/ferrdb/) collected 259 ferroptosis-related genes including driver, suppressor, and marker [18]. The confidence levels of genes involved in ferroptosis were assigned to 4 degrees including validated, screened, predicted, and deduced. The species involved included humans, mice, rats, and drosophila. To ensure the accuracy and stability of the model, 121 human-related and validated ferroptosis-related genes were obtained and provided in Supplementary Table S1.

### 2.2. Identification of Differentially Expressed and Prognostic Genes

In the primary cohort, ferroptosis-related genes with differential expression between tumor tissues and adjacent tissues were identified utilizing the “limma” R package according to the following cut-off value: false discovery rate (FDR) < 0.05. Through univariate Cox analysis, the association between expression levels of ferroptosis-related genes and GC patients' overall survival (OS) was explored. Overlapping ferroptosis-related genes with differential expression and prognostic value in the primary cohort were subjected to construct a prognostic model.

### 2.3. Construction and Validation of the Prognostic Ferroptosis-Related Model

Based on the expression of prognostic DEGs and survival data, the LASSO Cox regression analysis by R package “glmnet” was performed to further select the most useful prognostic markers and the penalty regularization parameter lambda was chosen based on 10 cross-validations. Through multiplying the expression level of a gene by its corresponding Cox regression coefficient, the risk score for each patient was calculated using the following formula: risk score = e^sum (each gene's expression × corresponding coefficient)^. The patients were separated into high- and low-risk groups based on the median value of the risk score. The “Rtsne” package and the prcomp function in the “stats” package were used to perform the t-SNE and PCA analysis to explore the distribution of high- and low-risk groups. Kaplan–Meier survival curves and a time-dependent ROC curve analysis were applied to compare the survival between the above two groups and evaluate the model's predictive ability using the “survivalROC” package in R, respectively.

### 2.4. Functional Enrichment Analysis

The enrichment analysis of Gene Ontology (GO) and Kyoto Encyclopedia of Genes and Genomes (KEGG) was carried out according to the DEGs to explore different molecular mechanisms and between high- and low-risk patients by utilizing the “clusterProfiler” R package. The *P* values are adjusted using the BH method to control the FDR.

### 2.5. Calculation of Immune Score, Stromal Score, and ESTIMATE Score

ESTIMATE (Estimation of Stromal and Immune cells in Malignant Tumor tissues using expression) algorithm was used to evaluate the ratio of the immune-stromal component in the tumor microenvironment (TME) through utilizing “estimate” R package, which generates three scores including Immune Score (reflecting the level of immune cells infiltrations), Stromal Score (reflecting the presence of stroma), and ESTIMATE Score (reflecting the sum of both) [19]. The higher the respective score is, the larger the ratio of the corresponding component in TME exists.

### 2.6. Immune Cells Infiltration and Immune-Related Pathways between Two Risk Groups

The infiltrating level of 16 immune cells and the activity of 13 immune-related pathways in each GC patient were quantified using single-sample gene set enrichment analysis (ssGSEA) [20] in the “gsva” R package. The annotated gene set file is presented in Supplementary Table S2.

### 2.7. Cell Culture

GC cell line AGS and normal human gastric epithelial cell line GES-1 were purchased from the American Type Culture Collection (ATCC, Manassas, VA, USA). All cells were cultured in RPMI-1640 Medium (Life Technologies, Grand Island, NY, USA) supplemented with 10% fetal bovine serum (Life Technologies) at 37°C in a humidified atmosphere with 5% CO2.

### 2.8. Quantitative Real-Time PCR

Total RNA was extracted from cells with TRIzol reagent (Invitrogen, China) in accordance with manufacturer's protocol. Reverse transcription was carried out according to the manufacturer's instructions using the PrimeScript RT Reagent Kit (Takara, China). The SYBR PrimeScript RT-PCR Kit (Takara) was applied for the analysis of quantitative reverse transcription-polymerase chain reaction (qRT-PCR). The 2^−ΔΔCt^ statistic was used to calculate the expression levels of genes. The concrete sequences of different primers used in this study are included in Supplementary Table S3.

### 2.9. Statistical Analysis

Student's *t*-test was applied to identify the differentially expressed ferroptosis-related genes between tumor tissues and adjacent tissues and evaluate the difference of Immune Score, Stromal Score, and ESTIMATE Score between risk groups. The Chi-squared test was used to compare the difference of proportion composition. The difference in ssGSEA scores of immune cells or pathways between the risk groups was evaluated by the Mann–Whitney test with *P* values adjusted by the BH method. The OS between groups was compared by using the Kaplan–Meier analysis with the log-rank test. And the identification of independent predictors of OS was conducted by the analysis of univariate and multivariate Cox regression. All statistical analyses were performed with R software (Version 3.6.3) or GraphPad Prism software (Version 8.0). All *P* values are two-tailed with a *P* value less than 0.05 was considered statistically significant.

## 3. Results

### 3.1. Identification of Prognostic Ferroptosis-Related DEGs in the TCGA Cohort

**Figure 2:**
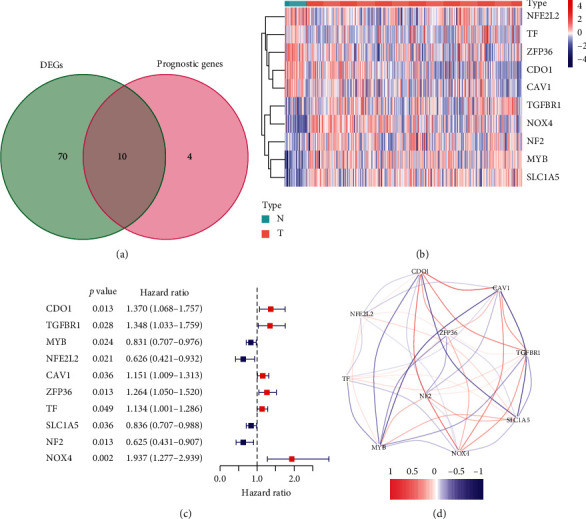
Identification of the ferroptosis-related genes with differential expression and prognostic value in the TCGA cohort. (a) Venn diagram showing differently expressed genes between tumor and adjacent tissues that were related to OS. (b) Heatmap showing the expression of the 10 overlapping genes between tumor and adjacent tissues. (c) Forest plots to show the results of the univariate Cox regression analysis between gene expression and OS. (d) The correlation network of the 10 overlapping genes. The correlation coefficients are distinguished by different colors.

A total of 80 DEGs were identified related to ferroptosis in GC, and 10 of them were correlated with OS (Figure 2(a)). Among the 10 prognostic ferroptosis-related DEGs, upregulated and downregulated genes accounted for half in tumor tissue, which was visualized using a heatmap (Figure 2(b)). According to the univariate Cox regression analysis, all of the 10 genes were significantly associated with the OS of GC patients, of which 6 were risk genes (HR > 1) and 4 were protective genes (HR < 1) (Figure 2(c)). The correlation between the above 10 prognostic ferroptosis-related DEGs is shown in Figure 2(d).

### 3.2. Construction of a Prognostic Model in the TCGA Cohort

**Figure 3:**
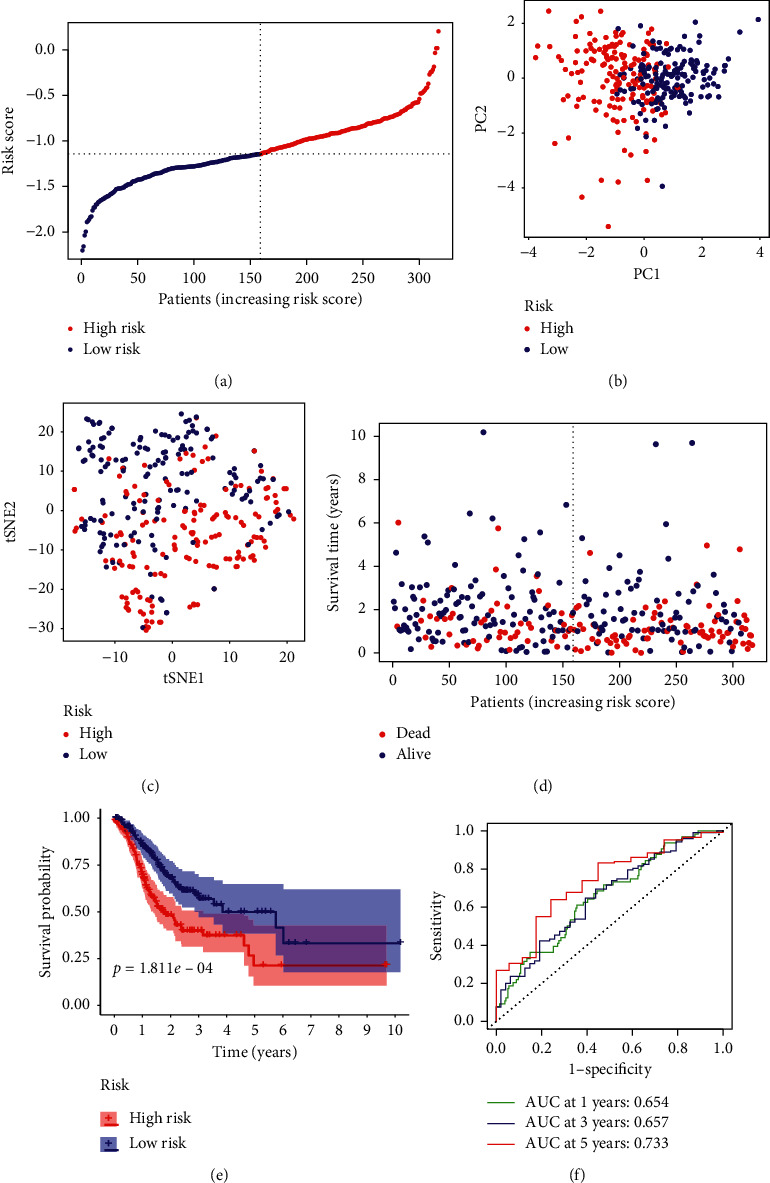
Prognostic analysis of the 8-gene model in the TCGA cohort. (a) The distribution of risk score in the TCGA cohort. (b) PCA analysis of the TCGA cohort. (c) T-SNE plot of the TCGA cohort. (d) The distribution of OS in the TCGA cohort. (e) The Kaplan–Meier survival analysis of OS between the high-risk group and low-risk group in the TCGA cohort. (f) AUC in ROC analysis for risk signature at 1-, 3- and 5-year survival time in the TCGA cohort.

Through LASSO Cox regression analysis, 8 predictors most contributing to the OS of GC patients were screened out (i.e., TCFBR1, MYB, NFE2L2, ZFP36, TF, SLC1A5, NF2, and NOX4) based on the optimal value of *λ* (Figure S1) and were subjected to construct a ferroptosis-related prognostic model using the following formula: risk score = *e*^(0.062^^*∗*^^expression level of TGFBR1 + −0.017^^*∗*^^expression level of MYB + −0.236^^*∗*^^expression level of NFE2L2 + 0.104^^*∗*^^expression level of ZFP36 + 0.112^^*∗*^^expression level of TF + −0.076^^*∗*^^expression level of SLC1A5 + −0.306^^*∗*^^expression level of NF2 + 0.462^^*∗*^^expression level of NOX4)^. Utilizing the median risk score as the cut-off value, the patients in the TCGA primary cohort were stratified into a high-risk group (*n* = 158) or a low-risk group (*n* = 159) (Figure 3(a)). The chi-squared test indicated that the higher risk group had a higher proportion of advanced tumor grade and stage in the TCGA cohort (Table 2). PCA and t-SNE analysis revealed that the patients in different risk groups were divided into two directions (Figures 3(b) and 3(c)). As presented in Figure 3(d), it indicated that patients of high-risk group possess a poor survival. The Kaplan–Meier survival analysis also confirmed the high-risk group yielding reduced survival time (Figure 3(e), *P* < 0.001). The time-dependent ROC curves were utilized to make an evaluation of the predictive performance of the model and the area under the curve (AUC) reached 0.654 at 1 year, 0.657 at 3 years, and 0.733 at 5 years (Figure 3(f)).

### 3.3. Validation of the Prognostic Model in the Meta-GEO Cohort

**Figure 4:**
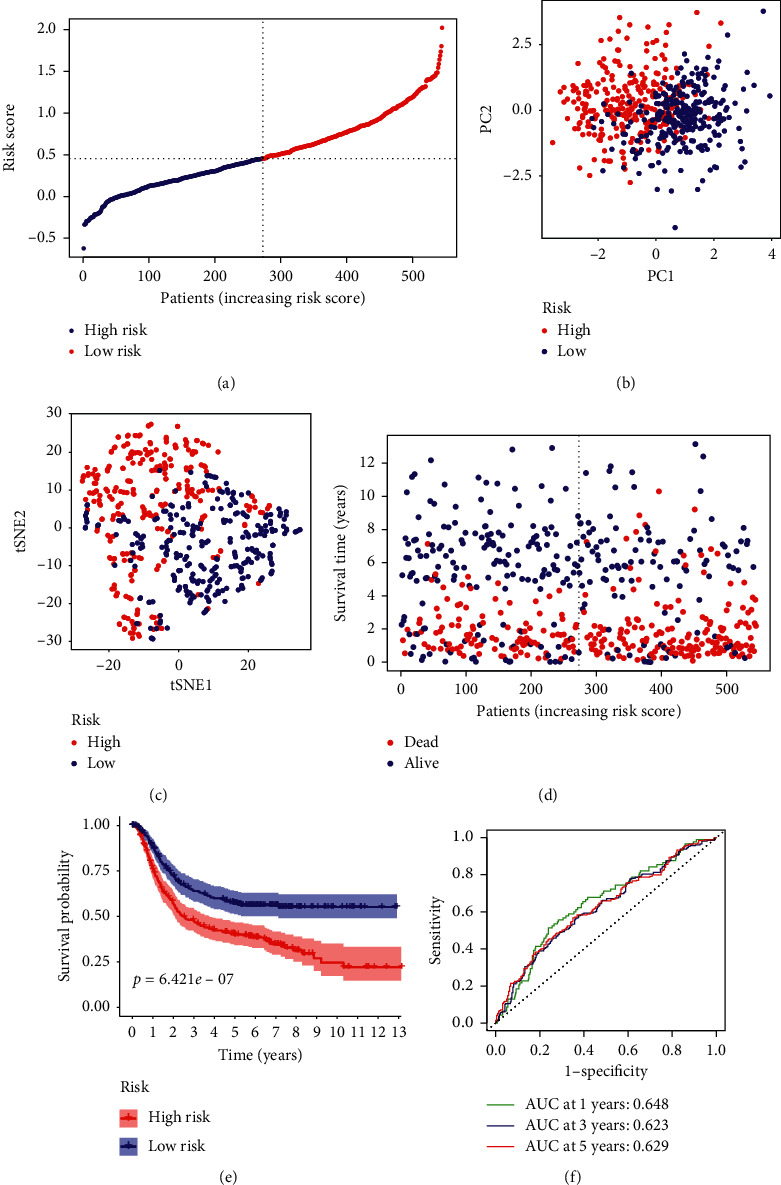
Validation of the prognostic model in the meta-GEO cohort. (a) The distribution of risk score in the meta-GEO cohort. (b) PCA analysis of the meta-GEO cohort. (c) T-SNE plot of the meta-GEO cohort. (d) The distribution of OS in the meta-GEO cohort. (e) The Kaplan–Meier survival analysis of OS between the high-risk group and low-risk group in the meta-GEO cohort. (f) AUC in ROC analysis for risk signature at 1-, 3- and 5-year survival time in the meta-GEO cohort.

In order to test the robustness of the model developed by the TCGA cohort, we calculated the risk score of each patient in the meta-GEO cohort by the same formula we obtained from the TCGA cohort. Then, according to the median value of the prognostic signature score, the patients from the meta-GEO cohort were divided into high-risk (*n* = 272) or low-risk groups (*n* = 273) as well (Figure 4(a)). In the meta-GEO cohort, the high-risk group also had a higher proportion of advanced tumor stage (Table 2). PCA and t-SNE analysis also confirmed a reliable clustering ability of risk score (Figures 4(b) and 4(c)). Similarly, patients in the high-risk group tended to suffer an earlier death and have a significantly shorter survival time than the low-risk group (Figure 4(d), *P* = 0.001). In addition, ROC analysis was performed, with the AUC values of 1, 3, and 5 years being 0.646, 0.623, and 0.629, respectively.

### 3.4. Independent Prognostic Value of the Risk Score

**Figure 5:**
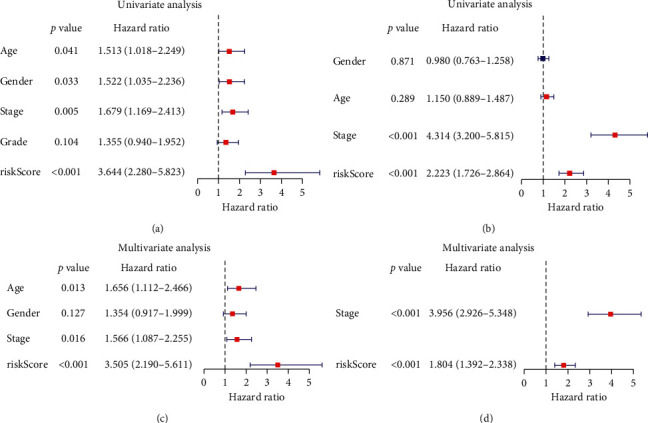
Risk score is an independent prognostic signature for GC. Results of the univariate and multivariate Cox regression analyses of OS in the TCGA cohort (a, c) and in the meta-GEO cohort (b, d).

To further explore whether the risk score was an independent prognostic factor, univariate and multivariate Cox regression analyses were carried out. In univariate Cox regression analyses, the risk score had a significant relationship with OS both in the TCGA cohort (HR = 3.644, 95% CI = 2.280–5.823, *P* < 0.001, Figure 5(a)) and the meta-GEO cohort (HR = 2.223, 95% CI = 1.726–2.864, *P* < 0.001, Figure 5(b)). As for the multivariate Cox regression analysis where potentially confounding factors were corrected, it indicated similarly that the risk score could serve as an independent predictor for OS (TCGA cohort: HR = 3.505, 95% CI = 2.190–5.611, *P* < 0.001; GEO cohort: HR = 1.804, 95% CI = 1.392–2.338, *P* < 0.001; Figures 5(c) and 5(d)).

### 3.5. Functional Analyses in the TCGA and the Meta-GEO Cohort

**Figure 6:**
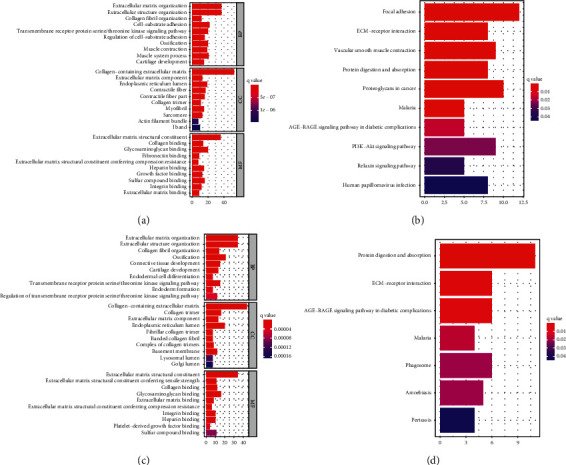
Functional enrichment of the DEGs between risk groups. The results of GO enrichment in the TCGA cohort (a) and in the meta-GEO cohort (c). The results of KEGG enrichment in the TCGA cohort (b) and in the meta-GEO cohort (d).

To clarify the biological functions and pathways correlated with the risk score, the enrichment analysis of GO enrichment and KEGG pathway was implemented based on the DEGs between the high-risk and low-risk groups in TCGA-STAD and meta-GEO cohort. According to GO enrichment analysis, the DEGs between risk groups from the TCGA and meta-GEO cohorts were mainly enriched in extracellular matrix (ECM) organization (*P*. adjust <0.05, Figures 6(a) and 6(c)). KEGG pathway analysis also showed that the ECM-receptor interaction pathway was significantly enriched in both cohorts (*P*. adjust <0.05, Figures 6(b) and 6(d)).

### 3.6. Estimation of the Proportion of Immune-Stromal Component

**Figure 7:**
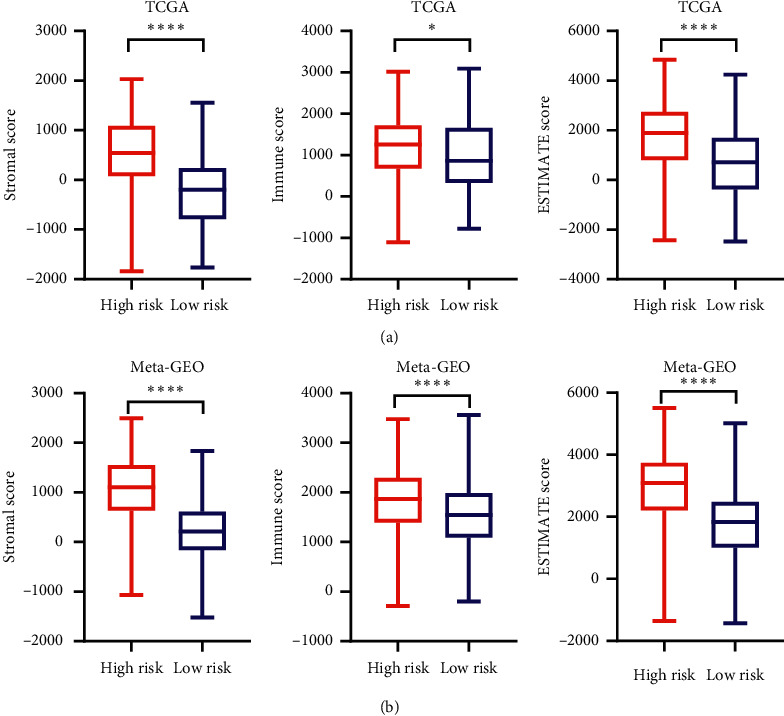
Estimation of the proportion of immune-stromal component. Immune Score, Stromal Score, and ESTIMATE Score (the sum of them) between different risk groups in the TCGA cohort (a) and in the meta-GEO cohort (b). ^*∗*^*P* < 0.05; ^*∗∗*^*P* < 0.01; ^*∗∗∗*^*P* < 0.001; ^*∗∗∗∗*^*P* < 0.0001.

GO and KEGG enrichment analyses illustrated that the differential functions and pathways between risk groups were mainly concentrated on extracellular matrix organization and ECM-receptor interaction pathway. Therefore, it is necessary to further explore the contents of the immune-stromal components in TME. According to the ESTIMATE algorithm, Immune Score, Stromal Score, and ESTIMATE Score (the sum of them) were significantly higher in high-risk groups (*P* < 0.05, Figures 7(a) and 7(b)) indicating that there exist more immune-stromal components in TME of the high-risk groups.

### 3.7. Immune Cells Infiltration and Immune-Related Pathways

**Figure 8:**
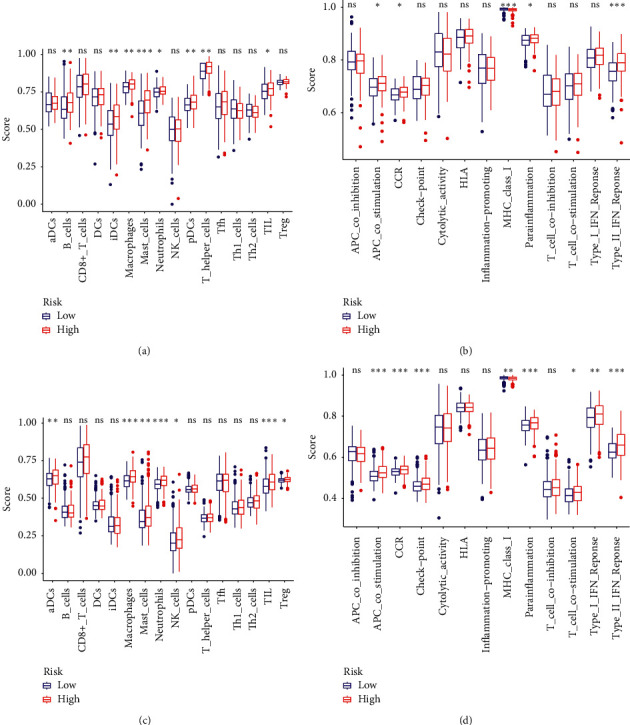
Differences of immune cells infiltration and immune-related pathways between risk groups. ssGSEA scores of 16 immune cells (a) and 13 immune-related functions (b) in the TCGA cohort. ssGSEA scores of 16 immune cells (c) and 13 immune-related functions (d) in the meta-GEO cohort. ns: not significant; ^*∗*^*P* < 0.05; ^*∗∗*^*P* < 0.01; ^*∗∗∗*^*P* < 0.001; ^*∗∗∗∗*^*P* < 0.0001.

To further discuss the differences in immune status between high- and low-risk groups, we estimated the enrichment scores of immune cells and immune-related functional pathways. In the TCGA cohort, the immune cell subpopulations of B cells, iDCs, macrophages, mast cells, neutrophils, pDCs, T helper cells, and TIL were significantly upregulated in the high-risk groups (all adjusted *P* < 0.05, Figure 8(a)). As for the immune-related pathways, APC costimulation, CCR, parainflammation, and type II IFN response were significantly upregulated in the high-risk group, while MHC class I was opposite (all adjusted *P* < 0.05, Figure 8(b)). Combined with the meta-GEO cohort, the alterations of macrophages, mast cells, neutrophils, TIL, APC costimulation, CCR, parainflammation, type II IFN response, and MHC class I were proved (all adjusted *P* < 0.05, Figures 8(c) and 8(d)).

### 3.8. Validation of Novel Genes Expression Levels in Cell Lines

**Figure 9:**
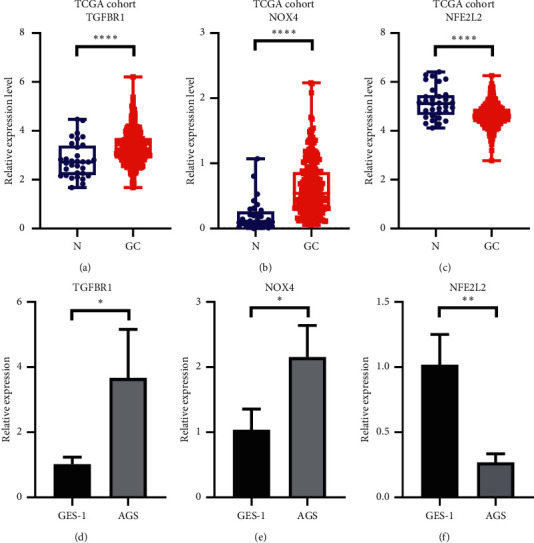
Differences of novel genes expression levels. Relative expression levels of (a) TGFBR1, (b) NOX4, and (c) NFE2L2 between adjacent tissues and GC tissues in the TCGA cohort. Relative expression levels of (d) TGFBR1, (e) NOX4, and (f) NFE2L2 between AGS and GES-1. ns: not significant; ^*∗*^*P* < 0.05; ^*∗∗*^*P* < 0.01; ^*∗∗∗*^*P* < 0.001; ^*∗∗∗∗*^*P* < 0.0001.

Among the 8 signatures, in the TCGA cohort, TGFBR1 and NOX4 were significantly upregulated in GC tissues (Figures 9(a) and 9(b), *P* < 0.0001) and harbored risk parameters HR > 1, suggesting they were novel oncogenes. NFE2L2 with HR<1 was significantly downregulated in GC tissues (Figure 9(c), *P* < 0.0001), suggesting it was a novel antioncogene. Their expression levels were evaluated in GES-1 and AGS by qRT-PCR. Consistently, compared with GES-1, TGFBR1 and NOX4 were significantly upregulated in AGS (Figures 9(d) and 9(e), *P* < 0.05) while NFE2L2 was remarkedly downregulated (Figure 9(f), *P* < 0.01).

## 4. Discussion

The heterogeneity of GC makes it important to develop stable prognostic indicators. In this study, we developed a ferroptosis-related model containing 8 signatures for predicting the prognosis of GC based on the data from TCGA and validated its predictive efficiency in a meta-GEO cohort. Both in the above two cohorts, patients with GC in the high-risk group harbored a shorter survival than those in the low-risk group. Functional analyses illustrated that the alteration of the risk score was mainly associated with extracellular matrix organization and ECM-receptor interaction pathway. Besides, it was also related to the characteristics of TME. Compared with the low-risk group, the high-risk group harbored higher Immune Score, Stromal Score, and ESTIMATE Score, suggesting that there existed higher levels in immune cells infiltration and stroma component but lower tumor purity in the TME of high-risk group. It is known that TME is typically characterized into three classes [21, 22]: (1) immune-inflamed: immune cells exist adjacent to tumor cells, (2) immune-excluded: immune cells exist around stroma but are not penetrating the tumor, (3) immune-desert: lacks immune cell infiltration. In the current study, according to the exhibitions of high-risk group with a higher abundance of immune cell infiltration and larger ratio of stroma component but poorer prognosis, it was reasonable to speculate that the TME of the high-risk group was in accordance with the immune-excluded subtype. In this context, although the TME displays an abundant infiltration of immune cells, they cannot effectively penetrate the tumor parenchyma to eliminate tumor cells. Therefore, it was not surprising that the high-risk group tended to carry a poorer prognosis.

We noticed that TIL were higher in the high-risk group while CD8+ T cell had no difference, suggesting that other types of cells in TIL may differ between risk groups. TIL are comprised of CD8+ T cells, CD4+ T cells, regulatory T cells, tumor associated macrophages, tumor associated neutrophils, myeloid derived suppressor cells, and natural killer cells, which interact with each other to exert antitumor or protumor effects [23]. Most cancers harbored a longer survival with a high amount of CD8+ T cells [24], while increased expression of protumor cells such as regulatory T cells, tumor associated macrophages, tumor associated neutrophils, and myeloid derived suppressor cells usually portend worse outcome [25]. In the current study, the high-risk group has a higher level of macrophages, mast cells, and neutrophils infiltration both in the TCGA and meta-GEO cohort. Studies have shown that tumor-associated macrophages, associated with poor prognosis of GC patients [26], can promote tumor cell proliferation [27], invasion [28], metastasis [29], and immune escape [30]. Increased levels of mast cells foster immune suppression and independently predict reduced OS of GC patients [31]. Tumor-infiltrating neutrophils can also restrain normal T-cell immunity and were associated with GC worse survival [32]. Apart from increased infiltration of protumor immune cells, high-risk groups also had a higher level of type II IFN response characterized by attenuated antitumor immunity. Overall, the enhancement of protumor immunity and the impairment of antitumor immunity may also be the cause of the poor prognosis.

The number of ferroptosis-related DEGs between GC and normal samples accounted for 66.1%, indicating a potential role of ferroptosis in GC. There were 8 signatures constituting the risk model, including TGFBR1, MYB, NFE2L2, ZFP36, TF, SLC1A5, NF2, and NOX4. According to the annotation from FerrDb (http://www.zhounan.org/ferrdb/) [18], TGFBR1, MYB, TF, SLC1A5, and NOX4 are drivers that promote ferroptosis, while NFE2L2, ZFP36, and NF2 are suppressors that prevent ferroptosis. They were reported to be involved in ferroptosis. TGFBR1, a type I receptor for TGF-*β*, can weaken erastin-induced HK-2 cell ferroptosis [33]. MYB is a well-known protooncogene protein which has been reportedly associated with the alterations of ROS [34]. In GC cells, inhibition of c-Myb can suppress the transcription of CDO1, therefore enhancing GSH generation, preventing ROS generation, and ultimately restraining erastin-induced ferroptosis. TF can transport iron into cells. Knockout of TF led to decreased ROS and ferroptosis [35]. SLC1A5 is a cell surface transporter responsible for the transport of neutral amino acids [36]. Overexpression of SLC1A5 can attenuate ferroptosis suppression mediated by miR-137 in melanoma cells. NOX4 participates in the generation of ROS and inhibition of it can block ferroptosis. NFE2L2, also namely NRF2, is mainly involved in the antioxidant response [37]. It served as the ferroptosis suppressor in multiple cancers including hepatocellular carcinoma [38], glioblastomas [39], head and neck cancer [40], and ovarian cancer [41]. ZFP36, regulating cell response to lipid peroxidation and oxidative stress, can lead to resistance to ferroptosis when overexpressed in liver fibrosis [42]. A recent study demonstrated that intercellular interaction between epithelial cells medicated by E-cadherin could suppress ferroptosis through Merlin-YAP signaling and inactivation of NF2, the Merin-encoding gene, enabled cancer cells to cause ferroptosis [43]. How these genes regulate ferroptosis and shape TME in GC remains to be further explored.

This study had some limitations. Firstly, our model was constructed and validated based on retrospective data. Prospective clinical validation is needed henceforth. Secondly, the correlation between risk score and TME in GC has not been investigated experimentally.

## 5. Conclusions

In conclusion, our study constructed a ferroptosis-related prognostic model for GC, which was an independent factor associated with OS. However, the underlying mechanisms between ferroptosis-related genes and TME in GC still remain to be explored.

## Figures and Tables

**Table 1:** Clinical characteristic of the GC patient used in this study.

	TCGA	GEO
No. of patients	317	545
Age (%)		
≤65	101 (31.9)	272 (49.9)
>65	216 (68.1)	274 (50.1)
Gender (%)		
Female	115 (36.3)	188 (34.4)
Male	202 (63.7)	358 (65.6)
Grade (%)		
G1	7 (2.2)	NA
G2	112 (35.3)	NA
G3	198 (62.5)	NA
Stage (%)		
I	17 (5.4)	72 (13.2)
II	67 (21.1)	137 (25.2)
III	150 (47.3)	187 (34.3)
IV	83 (26.2)	149 (27.3)
Survival status		
OS day (median)	479	956
Ending (%)		
Survival	188 (59.3)	273 (50.1)
Death	129 (40.7)	272 (49.9)

**Table 2:** Baseline characteristic of the patient in different risk group.

Characteristic	TCGA			GEO		
	High risk	Low risk	*P* value	High risk	Low risk	*P* value
Gender (%)			0.438137			0.610338
Female	54 (17.1)	61 (19.2)		91 (16.7)	97 (17.8)	
Male	104 (32.8)	98 (30.9)		181 (33.2)	176 (32.3)	

Age (%)			0.196178			0.023111
≤65	77 (24.3)	66 (20.8)		152 (27.9)	126 (23.1)	
>65	81 (25.6)	93 (29.3)		120 (22.0)	147 (27.0)	

Tumor grade (%)			0.0009			NA
G1 + G2	45 (14.2)	74 (23.3)		NA	NA	
G3	113 (35.7)	85 (26.8)		NA	NA	

Stage (%)			4.86*E* −* *06			0.000174
I + II	35 (11.0)	74 (23.3)		83 (15.2)	126 (23.1)	
III + IV	123 (38.9)	85 (26.8)		189 (34.7)	147 (27.0)	

## Data Availability

The data of this study are from TCGA and GEO databases.

## Supplementary Materials

Supplementary Materials Figure S1: construction of an 8-gene signature model in the TCGA cohort. (A) LASSO coefficient profiles of the expression of 10 overlapping genes. (B) Selection of the penalty parameter (*λ*) in the LASSO model via 10-fold cross-validation. Table S1: 121 human-related and validated ferroptosis-related genes. Table S2: the annotated gene set file used in ssGSEA. Table S3: the primers used in this study.
