# Supplementary material for: Construction and Validation of a Ferroptosis-Related Prognostic Model for Gastric Cancer
Source: J Oncol. 2021 Feb 28;2021:6635526. doi: 10.1155/2021/6635526 (PMC7937463; doi:10.1155/2021/6635526)
Supplement: Supplementary Materials — Figure S1: construction of an 8-gene signature model in the TCGA cohort. (A) LASSO coefficient profiles of the expression of 10 overlapping genes. (B) Selection of the penalty parameter (λ) in the LASSO model via 10-fold cross-validation. Table S1: 121 human-related and validated ferroptosis-related genes. Table S2: the annotated gene set file used in ssGSEA. Table S3: the primers used in this study. [file 6635526.f1.zip › 6635526.f1/Table S1.pdf]

Table S1. The ferroptosis-related genes.

RPL8
IREB2
ATP5MC3
CS
EMC2
ACSF2
G6PD
PGD
VDAC2
TP53
CARS1
KEAP1
HMOX1
ATG5
ATG7
NCOA4
TF
ALOX5
ALOX12
ALOX12B
ALOX15
ALOX15B
ALOXE3
PHKG2
ACSL4
SAT1
EGFR
NOX4
MAPK3
MAPK1
ZEB1
DPP4
CDKN2A
PEBP1
SOCS1
CDO1
MYB
SLC1A5
CHAC1
LINC00472
GOT1
BECN1
PRKAA2
PRKAA1
ELAVL1
BAP1
ABCC1
MIR6852
ACVR1B
TGFBR1
IFNG
ANO6
HMGB1
TNFAIP3
ATF3
ATM
YY1AP1

EGLN2
MIOX
TAZ
MTDH
IDH1
PANX1
LONP1
SLC7A11
GPX4
AKR1C1
AKR1C2
AKR1C3
RB1
HSPB1
HSF1
NFE2L2
SQSTM1
NQO1
FTH1
MUC1
MT1G
SLC40A1
CISD1
HSPA5
ATF4
HELLS
SCD
FADS2
SRC
STAT3
PML
NFS1
TP63
CDKN1A
MIR137
FH
CISD2
MIR9-1
MIR9-2
MIR9-3
CBS
ISCU
ACSL3
OTUB1
CD44
LINC00336
BRD4
PRDX6
MIR17
SESN2
NF2
ARNTL
HIF1A
CA9
TMBIM4
AIFM2
LAMP2
ZFP36

PROM2
CHMP5
CHMP6
CAV1
GCH1
PTGS2
