# Supplementary material for: Construction and Validation of a Ferroptosis-Related Prognostic Model for Gastric Cancer
Source: J Oncol. 2021 Feb 28;2021:6635526. doi: 10.1155/2021/6635526 (PMC7937463; doi:10.1155/2021/6635526)
Supplement: Supplementary Materials — Figure S1: construction of an 8-gene signature model in the TCGA cohort. (A) LASSO coefficient profiles of the expression of 10 overlapping genes. (B) Selection of the penalty parameter (λ) in the LASSO model via 10-fold cross-validation. Table S1: 121 human-related and validated ferroptosis-related genes. Table S2: the annotated gene set file used in ssGSEA. Table S3: the primers used in this study. [file 6635526.f1.zip › 6635526.f1/Table S2.pdf]

Table S2. The annotated gene set file used in ssGSEA.

aDCs	na	CD83	LAMP3	CCL1			
APC_co_inh	na	C10orf54	CD274	LGALS9	PDCD1LG2	PVRL3	
APC_co_sti	na	CD40	CD58	CD70	ICOSLG	SLAMF1	TNFSF14
B_cells	na	BACH2	BANK1	BLK	BTLA	CD79A	CD79B
CCR	na	CCL16	TPO	TGFBR2	CXCL2	CCL14	TGFBR3
CD8+_T_cel	na	CD8A					
Check-poir	na	IDO1	LAG3	CTLA4	TNFRSF9	ICOS	CD80
Cytolytic_	na	PRF1	GZMA				
DCs	na	CCL17	CCL22	CD209	CCL13		
HLA	na	HLA-E	HLA-DPB2	HLA-C	HLA-J	HLA-DQB1	HLA-DQB2
iDCs	na	CD1A	CD1E				
Inflammati	na	CCL5	CD19	CD8B	CXCL10	CXCL13	CXCL9
Macrophage	na	C1orf45	CD68	CLEC5A	CYBB	FUCA1	GPNMB
Mast_cells	na	CMA1	MS4A2	TPSAB1			
MHC_class_	na	B2M	HLA-A	TAP1			
Neutrophil	na	EVI2B	HSD17B11	KDM6B	MEGF9	MNDA	NLRP12
NK_cells	na	KLRC1	KLRF1				
Parainflam	na	CXCL10	PLAT	CCND1	LGMN	PLAUR	AIM2
pDCs	na	CLEC4C	CXCR3	GZMB	IL3RA	IRF7	IRF8
T_cell_co-	na	BTLA	C10orf54	CD160	CD244	CD274	CTLA4
T_cell_co-	na	CD2	CD226	CD27	CD28	CD40LG	ICOS
T_helper_c	na	CD4					
Tfh	na	PDCD1	CXCL13	CXCR5			
Th1_cells	na	IFNG	TBX21	CTLA4	STAT4	CD38	IL12RB2
Th2_cells	na	PMCH	LAIR2	SMAD2	CXCR6	GATA3	IL26
TIL	na	ITM2C	CD38	THEMIS2	GLYR1	ICOS	F5
Treg	na	IL12RB2	TMPRSS6	CTSC	LAPTM4B	TFRC	RNF145
Type_I_IFN	na	DDX4	IFIT1	IFIT2	IFIT3	IRF7	ISG20
Type_II_IF	na	GPR146	SELP	AHR			

TNFSF15	TNFSF18	TNFSF4	TNFSF8	TNFSF9			
FCRL1	FCRL3	HVCN1	RALGPS2				
IL11RA	CCL11	IL4I1	IL33	CXCL12	CXCL10	BMPER	BMP8A
PDCD1LG2	TIGIT	CD70	TNFSF9	ICOSLG	KIR3DL1	CD86	PDCD1
HLA-DQA2	HLA-DQA1	HLA-A	HLA-DMA	HLA-DOB	HLA-DRB1	HLA-H	HLA-B
GNLY	GZMB	IFNG	IL12A	IL12B	IRF1	PRF1	STAT1
HS3ST2	LGMN	MMP9	TM4SF19				
PADI4	SELL	TRANK1	VNN3				
MMP7	ICAM1	MX2	CXCL9	ANXA1	TLR2	PLA2G2D	ITGA2
LILRA4	PHEX	PLD4	PTCRA				
HAVCR2	LAG3	LAIR1	TIGIT				
SLAMF1	TNFRSF18	TNFRSF25	TNFRSF4	TNFRSF8	TNFRSF9	TNFSF14	
LTA	CSF2						
TIGIT	KLRD1	IRF4	PRKCQ	FCRL5	SIRPG	LPXN	IL2RG
NETO2	ADAT2	CHST2	CTLA4	NFE2L3	LIMA1	IL1R2	ICOS
MX1	MX2	RSAD2	TNFSF10				

CXCL11	IL21R	IL17B	TNFRSF9	ILF2	CX3CR1	CCR8	TNFSF12
LAIR1	TNFRSF8	TNFSF15	TNFRSF14	IDO2	CD276	CD40	TNFRSF4

HLA-DRB5	HLA-DOA	HLA-DPB1	HLA-DRA	HLA-DRB6	HLA-L	HLA-F	HLA-G
TBX21							

MX1	HMOX1	CD276	TIRAP	IL33	PTGES	TNFRSF12A	SCARB1

CCL5	LCK	TRAF3IP3	CD86	MAL	LILRB1	DOK2	CD6
HSDL2	HTATIP2	FKBP1A	TIGIT	CCR8	LTA	SLC35F2	IL21R

CSF3	TNFSF4	BMP3	CX3CL1	BMP5	CXCR2	TNFRSF10D	BMP2
TNFSF14	HHLA2	CD244	CD274	HAVCR2	CD27	BTLA	LGALS9
HLA-DMB	HLA-DPA1						

CD14	BLNK	IFIT3	RETNLB	IFIT2	ISG15	OAS2	REL

PAG1	LAX1	PLEK	PIK3CD	SLAMF1	XCL1	GPR171	XCL2
AHCYL1	SOCS2	ETV7	BCL2L1	RRAGB	ACSL4	CHRNA6	BATF

CXCL14	CCL28	CXCL3	BMP6	CCL21	CXCL9	CCL23	IL6
TMIGD2	CD28	CD48	TNFRSF25	CD40LG	ADORA2A	VTCN1	CD160

OAS3	CD44	PPARG	BST2	OAS1	NOX1	PLA2G2A	IFIT1

TBX21	CD2	CD53	KLHL6	SLAMF6	CD40	SIT1	TNFRSF4
LAX1	ADPRH	TNFRSF4	ANKRD10	CD274	CASP1	LY75	NPTN

TNFRSF18	IL17RD	IL17D	IL27	CCL7	IL1R1	CXCR4	CXCR2P1

CD44	TNFSF18	TNFRSF18	BTNL2	C10orf54	CD200R1	TNFSF4	CD200

IFITM3	IL1RN

CD79A	CD247	LCP2	CD3D	CD27	SH2D1A	FYB	ARHGAP30
SSTR3	GRSF1	CSF2RB	TMEM184C	NDFIP2	ZBTB38	ERI1	TRAF3

TGFB1I1	IFNGR1	IL9R	IL1RAPL1	IL11	CSF1	IL20RA	IL25
NRP1							

ACAP1	CST7	CD3G	IL2RB	CD3E	FCRL3	CORO1A	ITK
NAB1	HS3ST3B1	LAYN	JAK1	VDR	LEPROT	GCNT1	PTPRJ

TNFRSF4	IL18	ILF3	CCL20	TNFRSF12A	IL6ST	CXCL13	IL12B

TCL1A	CYBB	CSF2RB	IKZF1	NCF4	DOCK2	CCR2	PTPRC
IKZF2	CSF1	ENTPD1	TNFRSF18	METTL7A	KSR1	SSH1	CADM1

TNFRSF8	IL6R	BMPR2	IFNE	IL1RAPL2	IL3RA	BMP4	CCL24

PLAC8	NCKAP1L	IL7R	6-Sep	CD28	STAT4	CD8A	LY9
IL1R1	ACP5	CHST7	THADA	CD177	NFAT5	ZNF282	MAGEH1

TNFSF13B	CCR4	IL2RA	IL32	TNFRSF10C	IL22RA1	BMPR1A	CXCR5
CD48	HCST	PTPRCAP	SASH3	ARHGAP25	LAT	TRAT1	IL10RA

CXCR3	IFNA8	IL17REL	IFNB1	IFNAR1	TNFRSF1B	CCL17	IFNL1
PAX5	CCR7	DOCK11	PARVG	SPNS1	CD52	HCLS1	ARHGAP9

IL16	IL1RL1	ILK	CCL25	ILDR2	CXCR1	IL36RN	IL34

GIMAP6	PRKCB	MS4A1	GPR18	TBC1D10C	GVINP1	P2RY8	EVI2B

TGFB1	IFNG	IL19	ILKAP	BMP2K	CCR10	ILDR1	EPO
VAMP5	KLRK1	SELL	MPEG1	MS4A6A	ARHGAP15	MFNG	GZMK

CCR7	IL17C	IL23A	CCR5	IL7	EPOR	CCL13	IL2RG
SELPLG	TARP	GIMAP7	FAM65B	INPP5D	ITGA4	MZB1	GPSM3

IL31RA	TNFAIP6	IFNL2	BMP1	IL12RB1	TNFAIP8	IL4R	TNFRSF6B
STK10	CLEC2D	IL16	NLRC3	GIMAP5	GIMAP4	IFFO1	CFH

TNFAIP8L1 TNFRSF10B IFNL3 CCL5 CXCL6 CXCL1 CCR3 TNFSF11

PVRIG CFHR1

CSF1R	IL21	IL1RAP	IL12RB2	CCL1	IL17RA	CCR1	IL1RN

TNFRSF11B	TNFRSF14	IL13	IL2RB	BMP8B	CCL2	IL24	IL18RAP

TGFBI	TNFSF10	TNFRSF11A	CXCL5	IL5RA	TNFSF9	IL1RL2	TNFRSF13C

IL36G	IL15RA	TNFRSF21	CXCL8	IL22RA2	TNFAIP8L2	IL18R1	IFNLR1

CXCR6	CCL3L3	TNFRSF1A	IL17RE	IFNGR2	IL17RC	TNFAIP8L3	ILVBL

TGFBRAP1	CCL4L1	CSF2RA	CCRN4L	CCL26	TNFAIP1	CCRL2	IFNA10

TNFRSF17	IFNA13	IL20	IL18BP	CCL3L1	TNFSF12-TN	IL5	IL23R

IL26	TNF	TGFA	CSF2	IL1F10	CXCL17	TNFSF13	IFNA4

IL37	IL12A	IL7R	IFNA1	IL1A	IL4	IL2	CCL22

CSF3R	IL10	IFNK	TGFB2	IL1R2	IL1B	IL17F	IL27RA

IL15	TNFSF8	IL36B	XCL1	CXCL16	TNFRSF19	IL3	CCL3

IFNA2	BMPR1B	IFNA21	TNFSF18	CCL8	IL17RB	TNFRSF25	IL22

IL10RB	IFNAR2	CCL18	IFNA16	CSF2RB	IL36A	TNFAIP3	IL13RA2

IL13RA1	CCR9	TNFRSF10A	IFNA7	IFNW1	XCL2	TNFSF14	CCR2

BMP15	BMP10	CCL15-CCL1	TGFBR1	IFNA5	BMP7	IFNA14	IL20RB

IL10RA	IFNA17	CCR6	TGFB3	CCL15	CCL4	CCL27	TNFRSF13B
