# Supplementary figures and images for: Construction and Validation of a Ferroptosis-Related Prognostic Model for Gastric Cancer
Source: J Oncol. 2021 Feb 28;2021:6635526. doi: 10.1155/2021/6635526 (PMC7937463; doi:10.1155/2021/6635526)

A

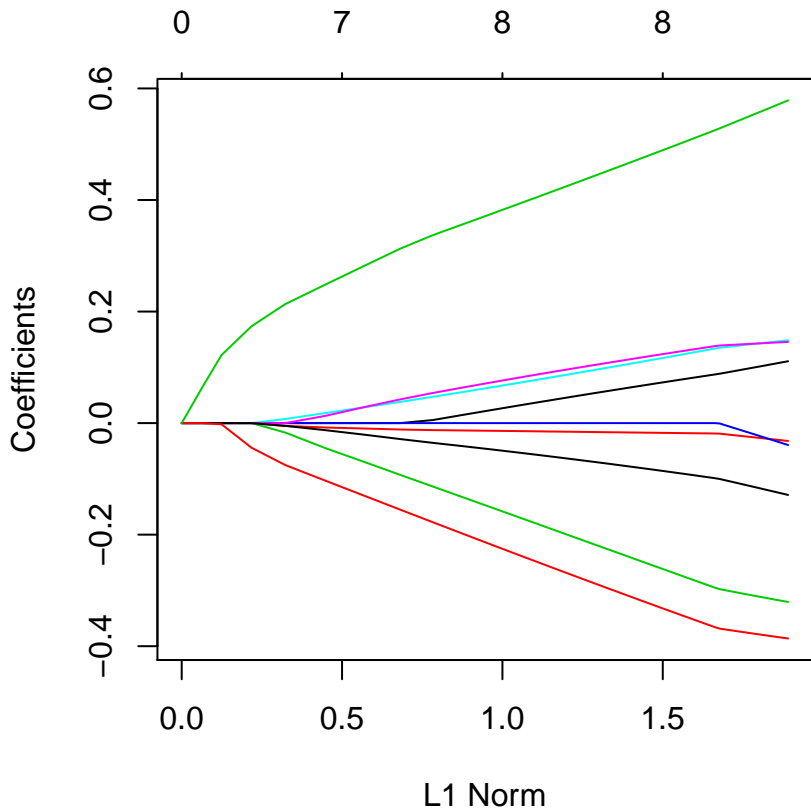

B

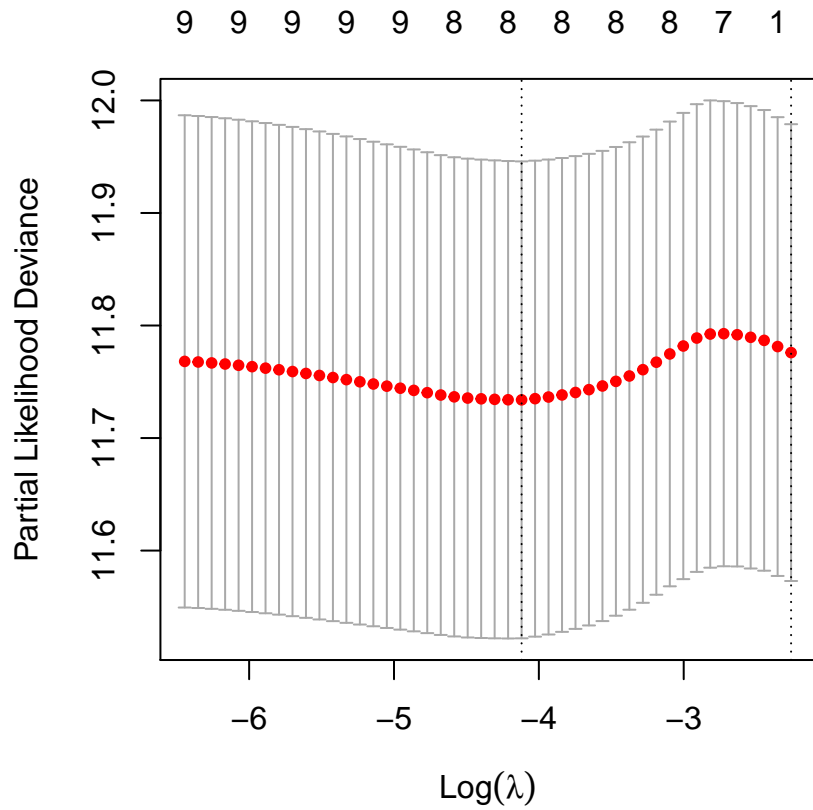

Supplement: Supplementary Materials — Figure S1: construction of an 8-gene signature model in the TCGA cohort. (A) LASSO coefficient profiles of the expression of 10 overlapping genes. (B) Selection of the penalty parameter (λ) in the LASSO model via 10-fold cross-validation. Table S1: 121 human-related and validated ferroptosis-related genes. Table S2: the annotated gene set file used in ssGSEA. Table S3: the primers used in this study. [file 6635526.f1.zip › 6635526.f1/Figure S1.pdf]
